# Supplementary material for: Zygotic pioneer factor activity of Odd-paired/Zic is necessary for late function of the Drosophila segmentation network
Source: eLife. 2020 Apr 29;9:e53916. doi: 10.7554/eLife.53916 (PMC7190358; doi:10.7554/eLife.53916)
Supplement: Source code 1. — This file is a markdown document that shows how critical aspects of the analysis described in this paper was performed. It is intended to provide transparency on how this was done from both a scientific and a methodological standpoint. We note that both this document, as well as all input data files necessary for reproducing this analysis are provided on the lab’s github page https://github.com/sblythe/Patternless_ATAC. [file elife-53916-code1.zip › Analysis_markdown.html]

WT versus Patternless Embryo ATAC Analysis


# WT versus ‘Patternless’ Embryo ATAC Analysis

#### Shelby Blythe

#### 11/22/2019

## Introduction

ATAC seq was performed on single wild-type or *bcd osk cic tsl TlRM9* embryos collected at either 12 or 72 minutes following anaphase 13 (NC14 + 12 or + 72), three embryos each per timepoint/genotype, twelve embryos total. Peaks were called on mapped reads of all samples pooled together. The analysis below performs DESeq2 on the mapped reads to find regions that are differentially enriched either over time or between genotypes. Different categories of peaks are defined based on the DESeq2 analysis that define classes of peaks that undergo changes in chromatin accessibility uniformly (i.e., independently of genotype), or in likely distinct subsets of cells based on inputs from maternal patterning systems (i.e., dependent on genotype). Included is code that exports the genome sequence of peaks within each class, as well as a background set for feeding to MEME for the purposes of identifying enriched motifs. Similarly, a representative region from each class is plotted using the GViz package.

This markdown also contains the comparison with different ChIP-seq and ATAC-seq datasets (Opa, maternal-Opa, Bcd, Zld). This is mainly intended to allow for a single consistent peaks list and analysis output to be made.

## Setup

Packages for the analysis are loaded below. Also, input and output file paths are designated in this section. This should be the only section that requires user input.

```
rm(list = ls())
gc()  # clear the workspace and perform garbage collection to free up any memory tied up in previous R tasks.
```

```
##          used (Mb) gc trigger (Mb) limit (Mb) max used (Mb)
## Ncells 468581 25.1    1023228 54.7         NA   631148 33.8
## Vcells 925854  7.1    8388608 64.0      65536  1761009 13.5
```

```
suppressPackageStartupMessages({
    library(DESeq2)
    library(GenomicRanges)
    library(BiocParallel)
    library(IHW)
    library(ggplot2)
    library(Gviz)
    library(Biostrings)
    library(BSgenome.Dmelanogaster.UCSC.dm6)
    library(TxDb.Dmelanogaster.UCSC.dm6.ensGene)
    library(zoo)
})
```

```
# This should contain all of the variables that are system-specific and would
# need to be changed if this code were to be run on another machine. The
# exceptions to this are examples below where I've included code but do not have
# the markdown set to evaluate the code (e.g., exporting publication quality
# images). There are other variables in the code below that represent analysis
# 'choices' that I've hard-coded into the examples as a record of what choices
# were made in the analysis.

multicoreN = 8  # enter the number of cores you want to designate for the DESeq2 analysis

peakfile = "~/Dropbox/Blythe Lab/Patternless Paper/Patternless_ATAC/All_Samples_shortreads_q1e-5_MACS2_peaks.narrowPeak.GR"
metadatafile = "~/Dropbox/Blythe Lab/Patternless Paper/Patternless_ATAC/170914_ATAC_library_prep_metadata.R"
openreadsfile = "~/Dropbox/Blythe Lab/Patternless Paper/Patternless_ATAC/patternless_all_open_reads_list.GR"
openwigfile = "~/Dropbox/Blythe Lab/Patternless Paper/Patternless_ATAC/patternless_all_open_reads_merged_by_sample_basecount_list_export.GR"

wtreads = "~/Dropbox/Blythe Lab/Patternless Paper/Patternless_ATAC/Hannon_wild-type_NC14+12_open_list_dm6.gr"
bcdreads = "~/Dropbox/Blythe Lab/Patternless Paper/Patternless_ATAC/Hannon_bcdE1_NC14+12_open_list_dm6.gr"
zldreads = "~/Dropbox/Blythe Lab/Patternless Paper/Patternless_ATAC/Hannon_zld_NC14+12_open_list_dm6.gr"

opareadsfile1 = "~/Dropbox/Blythe Lab/Patternless Paper/Patternless_ATAC/opa_all_open_reads_list.GR"
opareadsfile2 = "~/Dropbox/Blythe Lab/Patternless Paper/Patternless_ATAC/opa_all_open_reads_pool2_list.GR"
opametafile = "~/Dropbox/Blythe Lab/Patternless Paper/Patternless_ATAC/171117_ATAC_library_prep_data.txt"

opaChIPpeaksfile = "~/Dropbox/Blythe Lab/Patternless Paper/Patternless_ATAC/opa.ChIP.peaks_IDR_filtered.GR"

tubATACreads = "~/Dropbox/Blythe Lab/Patternless Paper/Patternless_ATAC/tub-opa_all_open_reads_list.R"
wt13ATACreads = "~/Dropbox/Blythe Lab/Patternless Paper/Patternless_ATAC/wt_NC13+12_all_open_reads_list.R"
tubmetafile = "~/Dropbox/Blythe Lab/Patternless Paper/Patternless_ATAC/190813_ATAC_library_prep_metadata.txt"

sequence.output.directory = "~/Dropbox/Blythe Lab/Patternless Paper/Patternless_ATAC/Peak_Class_Sequences"
```

## DESeq2 analysis

Next we set up the `DESeq2` analysis:

```
register(MulticoreParam(multicoreN))

# chromosomes of interest

good.uns = c("chr2L", "chr2R", "chr3L", "chr3R", "chr4", "chrX")

# load peaks

load(peakfile)  #peaks

# load the metadata

load(metadatafile)  #meta

# load the open reads

load(openreadsfile)  #allopen

# name the peaks

names(peaks) = paste0(seqnames(peaks), ":", start(peaks), "-", end(peaks))

# filter peaks by those within chromosomes of interest:

peaks = peaks[seqnames(peaks) %in% good.uns]

# set aside the original peaks list prior to further filtering:

orig = peaks

# remove all peaks that have a q-score greater than 1e-15

peaks = peaks[score(peaks) >= 15]

# count the number of small ATAC fragments that overlap with a peak

counts = lapply(allopen, function(x) countOverlaps(peaks, x))
counts = do.call("cbind", counts)  # forces the counts data into a table format where rows = peaks and columns = samples

# generate the column data from the metadata file

coldata = meta
rownames(coldata) = paste0(coldata[, 2], "_", coldata[, 3], "_", coldata[, 1])  # generate a vector that matches the prior names assigned to each ATAC sample
# the metadata table needs to be streamlined for the DESeq analysis
geno = rep("mut", nrow(coldata))  # make a new vector with simple genotypes
geno[coldata$genotype == "wt"] = "wt"
coldata = coldata[, -1]  # lose the first column which is now redundant with the rownames
coldata = coldata[, c(1:4, 6, ncol(coldata))]  # keep the columns that could be of potential interest in this analysis.
coldata[, 1] = geno  # replace the complex genotype column with our simplified one
colnames(coldata)[2] = "time"  # rename the second column
condition = paste0(geno, "_", coldata[, 2])  # make a new vector that combines all useful identifier features in case we want it.
condition = gsub("NC14[+]", "", condition)  # trim away the redundant information
coldata[, 2] = gsub("NC14[+]", "", coldata[, 2])  # clean up the 'time' column as well
coldata = cbind(condition, coldata)  # append the 'condition' vector
coldata[, 4] = round(coldata[, 4])
for (i in 1:ncol(coldata)) {
    coldata[, i] = as.factor(coldata[, i])
}  # convert all columns to 'factor'

# create an ordering vector:
orderer = c(which(coldata[, 1] == "wt_12"), which(coldata[, 1] == "wt_72"), which(coldata[, 
    1] == "mut_12"), which(coldata[, 1] == "mut_72"))

# reorder the counts table:
counts = counts[, orderer]

# reorder the coldata table:
coldata = coldata[orderer, ]

# make sure the intended reference factor levels are the first factor levels
coldata[, 1] = relevel(coldata[, 1], ref = "wt_12")
coldata[, 2] = relevel(coldata[, 2], ref = "wt")
coldata[, 3] = relevel(coldata[, 3], ref = "12")
# The others are ok to leave as the minimum value

# check that everything is in register:
all(rownames(coldata) %in% colnames(counts))
```

```
## [1] TRUE
```

```
all(rownames(coldata) == colnames(counts))
```

```
## [1] TRUE
```

We can visually check that everything looks ok.

```
coldata
```

```
##                          condition genotype time collection.temp
## wt_NC14+12_17091303          wt_12       wt   12              22
## wt_NC14+12_17091304          wt_12       wt   12              23
## wt_NC14+12_17091306          wt_12       wt   12              22
## wt_NC14+72_17091301          wt_72       wt   72              22
## wt_NC14+72_17091302          wt_72       wt   72              22
## wt_NC14+72_17091501          wt_72       wt   72              22
## boctRM9_NC14+12_17091103    mut_12      mut   12              22
## boctRM9_NC14+12_17091202    mut_12      mut   12              22
## boctRM9_NC14+12_17091203    mut_12      mut   12              22
## boctRM9_NC14+72_17091102    mut_72      mut   72              22
## boctRM9_NC14+72_17091201    mut_72      mut   72              22
## boctRM9_NC14+72_17091305    mut_72      mut   72              23
##                          library.prep.date PCR.cycles sex
## wt_NC14+12_17091303                9/13/17         11   f
## wt_NC14+12_17091304                9/13/17         11   m
## wt_NC14+12_17091306                9/13/17         11   f
## wt_NC14+72_17091301                9/13/17         11   f
## wt_NC14+72_17091302                9/13/17         11   m
## wt_NC14+72_17091501                9/15/17         11   m
## boctRM9_NC14+12_17091103           9/11/17         13   m
## boctRM9_NC14+12_17091202           9/12/17         12   m
## boctRM9_NC14+12_17091203           9/12/17         10   f
## boctRM9_NC14+72_17091102           9/11/17         11   f
## boctRM9_NC14+72_17091201           9/12/17         10   m
## boctRM9_NC14+72_17091305           9/13/17         11   f
```

```
head(counts)
```

```
##                   wt_NC14+12_17091303 wt_NC14+12_17091304
## chr2L:5505-6046                   119                 178
## chr2L:6585-6881                    53                  72
## chr2L:7521-7933                    43                  51
## chr2L:17045-17167                  12                  22
## chr2L:17535-17617                  12                  19
## chr2L:18288-18359                   8                  14
##                   wt_NC14+12_17091306 wt_NC14+72_17091301
## chr2L:5505-6046                   149                  51
## chr2L:6585-6881                    49                  32
## chr2L:7521-7933                    57                  36
## chr2L:17045-17167                  14                  25
## chr2L:17535-17617                  19                  22
## chr2L:18288-18359                  10                  16
##                   wt_NC14+72_17091302 wt_NC14+72_17091501
## chr2L:5505-6046                   104                  98
## chr2L:6585-6881                    30                  40
## chr2L:7521-7933                    68                  71
## chr2L:17045-17167                  41                  49
## chr2L:17535-17617                  41                  38
## chr2L:18288-18359                  35                  33
##                   boctRM9_NC14+12_17091103 boctRM9_NC14+12_17091202
## chr2L:5505-6046                        184                      202
## chr2L:6585-6881                         43                       68
## chr2L:7521-7933                         38                       46
## chr2L:17045-17167                       18                       26
## chr2L:17535-17617                       27                       36
## chr2L:18288-18359                       13                       12
##                   boctRM9_NC14+12_17091203 boctRM9_NC14+72_17091102
## chr2L:5505-6046                        116                       89
## chr2L:6585-6881                         34                       34
## chr2L:7521-7933                         31                       58
## chr2L:17045-17167                       16                       18
## chr2L:17535-17617                       20                       16
## chr2L:18288-18359                        5                       12
##                   boctRM9_NC14+72_17091201 boctRM9_NC14+72_17091305
## chr2L:5505-6046                        167                      137
## chr2L:6585-6881                         44                       63
## chr2L:7521-7933                         57                       42
## chr2L:17045-17167                       38                       41
## chr2L:17535-17617                       25                       21
## chr2L:18288-18359                       15                       19
```

This sets up the DESeq2 analysis, which we do in the next chunk.

```
# initialize the DESeq dataset and process it:

dds = DESeqDataSetFromMatrix(countData = counts, colData = coldata, design = ~genotype + 
    time + genotype:time)
dds = DESeq(dds, parallel = TRUE)
resultsNames(dds)  # check the output of the comparisons made based on the design above.
```

```
## [1] "Intercept"          "genotype_mut_vs_wt" "time_72_vs_12"     
## [4] "genotypemut.time72"
```

```
# designate the alpha level for significance testing:
alphalevel = 0.05

# get the results for each comparison of interest:

res.wt.time = results(dds, contrast = c("time", "72", "12"), filterFun = ihw, alpha = alphalevel)  # this will score peaks on the basis of whether they significantly change over time in the wild-type dataset. (i.e., whether time is different in the reference genotype)

res.12.geno = results(dds, contrast = c("genotype", "mut", "wt"), filterFun = ihw, 
    alpha = alphalevel)  # this will score peaks on the basis of whether they significantly change in the reference timepoint between genotypes. 

res.mut.time = results(dds, contrast = list(c("time_72_vs_12", "genotypemut.time72")), 
    filterFun = ihw, alpha = alphalevel)  # this will score peaks on the basis of whether they significantly change in the non-reference genotype over time. 

res.72.geno = results(dds, contrast = list(c("genotype_mut_vs_wt", "genotypemut.time72")), 
    filterFun = ihw, alpha = alphalevel)  # this will score peaks on the basis of whether they significantly change in the non-reference timepoint between genotypes.

# interaction = results(dds, name = 'genotypemut.time72', filterFun = ihw, alpha
# = alphalevel) # this will score peaks on the basis of whether there is an
# interaction between genotype and time (i.e., whether there is a different
# effect based on the reference levels of each parameter.). I have commented this
# out for the final version of this analysis since this comparison was not used.
```

The `res...` objects contain the results tables from the DESeq2 analysis. `res.wt.time` indicates scores for the comparison of the two timepoints in ‘wild-type’. `res.mut.time` indicates scores for the comparison of the two timepoints in ‘mutant’. `res.12.geno` indicates scores for the comparison of the early timepoint between genotypes. `res.72.geno` indicates scores for the comparison of the late timepoint between genotypes.

## Peak Classification

We now want to identify regions of interest and categorize their behaviors over time. This will be facilitated by creating a ‘normalized’ measurement of chromatin accessibility for each peak so that all peaks occupy a similar intensity range, and so that differences over time and between genotypes are relatively preserved. I’ve opted for normalizing the reads so that they all sum to a value of 1.

```
cts = counts(dds, normalized = TRUE)
cts = sapply(c(1:nrow(cts)), function(x) c(mean(cts[x, 1:3]), mean(cts[x, 4:6]), 
    mean(cts[x, 7:9]), mean(cts[x, 10:12])))
cts.norm = apply(cts, 2, function(x) x/sum(x))
colnames(cts.norm) = names(dds)
```

The object `cts.norm` contains the normalized counts. Below I append this and the output from the DESeq2 analysis to the peaks list.

In the original analysis of this data, I initially used a clustering based approach to explore how many different groupings of data were reasonable within this analysis. Although the different classes I ultimately settle on are not derived directly from this clustering-based analysis, it was useful for directing the class definitions that I do later. The code for an example of this follows below.

```
# if I take all of the peaks that have significant increases in both genotypes,
# how many clusters can I get?

cutoff = 1e-04

sig.names = rownames(dds)[which(res.wt.time$padj < cutoff | res.mut.time$padj < cutoff | 
    res.12.geno$padj < cutoff | res.72.geno$padj < cutoff)]

test = cts.norm[, which(colnames(cts.norm) %in% sig.names)]
padj = cbind(res.wt.time[rownames(res.wt.time) %in% sig.names, "padj"], res.mut.time[rownames(res.mut.time) %in% 
    sig.names, "padj"], res.12.geno[rownames(res.12.geno) %in% sig.names, "padj"], 
    res.12.geno[rownames(res.12.geno) %in% sig.names, "padj"])

padj2 = apply(padj, 2, rank)/nrow(padj)

ncuts = 10
set.seed(8)
k = kmeans(t(test), centers = ncuts, nstart = 20, algorithm = "Hartigan-Wong")
kk = fitted(k, method = "classes")

cuts = rep(0, length(peaks))
for (i in 1:length(table(kk))) {
    cuts[names(peaks) %in% names(kk)[kk == i]] = i
}
```

The different clusters are plotted below. Note that these are meant to give a sense of the different kinds of groups that can be pulled out of the data, but they do not take into account the statistical tests from DESeq2, except as an initial filtering to determine which peaks to focus on.

```
xx = c(0.75, 2.25, 2.75, 4.25)
ylimits = c(0, 0.75)

par(mar = c(2.5, 2.5, 1, 1))
layout(matrix(c(1:12), nrow = 2, byrow = TRUE))
plot(xx, apply(test, 1, mean), type = "n", xlim = c(0.5, 4.5), ylim = ylimits, xaxt = "n", 
    main = "all")
axis(1, at = c(1:4), labels = c("w12", "w72", "m12", "m72"), las = 2)
text(1, ylimits[2], paste0(length(kk)), cex = 0.8)
. = sapply(c(1:ncol(test)), function(x) {
    lines(xx[1:2], test[1:2, x], col = rgb(0, 0, 0, 0.05))
    lines(xx[3:4], test[3:4, x], col = rgb(0, 0, 0, 0.05))
})


for (i in 1:ncuts) {
    plot(xx, apply(test[, kk == i], 1, mean), xlim = c(0.5, 4.5), ylim = ylimits, 
        xaxt = "n", main = paste0("cut ", i), pch = 4, col = "red")
    text(1, ylimits[2], paste0(length(which(kk == i))), cex = 0.8)
    . = sapply(which(kk == i), function(x) {
        lines(xx[1:2], test[1:2, x], col = rgb(0, 0, 0, 0.05))
        lines(xx[3:4], test[3:4, x], col = rgb(0, 0, 0, 0.05))
    })
}
```

Another note: in the (years) that I’ve been messing around with this data, the cluster numbering (but not the types of clusters) has varied depending on what version of R packages I’ve used, as well as what operating system I’ve run this type of code on. As such, the clustering based approach is unreliable if the final output is a cluster number and there is interest in the analysis being independently reproducible on different computing systems. Partly because of this, and partly because I should be able to arrive at a similar set of groups by only examining the statistical tests from DESeq2, I have not kept the clustered groups as the final set of differential classes. Below, we seek to define peak classes entirely on the basis of the DESeq2 analysis.

In the next chunk, I append the output from DESeq2 to the peaks list, as well as the normalized counts. Having all this data summarized in one place is useful in downstream steps.

```
meta = mcols(peaks)
norm = t(cts.norm)
mcols(peaks) = cbind(meta, data.frame(norm.wt.12 = round(norm[, 1], 4), norm.wt.72 = round(norm[, 
    2], 4), norm.mut.12 = round(norm[, 3], 4), norm.mut.72 = round(norm[, 4], 4), 
    baseMean = round(res.wt.time$baseMean, 4), res.wt.time.FC = round(res.wt.time$log2FoldChange, 
        4), res.wt.time.padj = (res.wt.time$padj), res.mut.time.FC = round(res.mut.time$log2FoldChange, 
        4), res.mut.time.padj = (res.mut.time$padj), res.12.geno.FC = round(res.12.geno$log2FoldChange, 
        4), res.12.geno.padj = (res.12.geno$padj), res.72.geno.FC = round(res.72.geno$log2FoldChange, 
        4), res.72.geno.padj = (res.72.geno$padj)))
```

Then, we’ll have two classes that are uniformly open but are repressed late, either uniformly or dependent on patterning.

Next we’ll have pattern dependent early accessible regions, either constitutively open, or repressed late

Finally we’ll have two classes that open late, either uniformly, or dependent on patterning.

There are seven classes overall, and eleven once we take into account whether the patterning dependent events occur in the wild-type or the mutant class.

To define these peak regions, first off, the uniform early constitutive peaks will be all the regions that don’t have a significant change in accessibility over time or between genotypes.

We can define the set of non uniform early constitutive peaks as a set of dynamic peaks that fulfill the criteria of having at least one significant p-value and having a fold change (for that p-value) that is above threshold. These will be the dynamic peaks.

```
thresh = 0.01
fcthresh = 1

dynamic = peaks[which((peaks$res.12.geno.padj < thresh & abs(peaks$res.12.geno.FC) > 
    fcthresh) | (peaks$res.72.geno.padj < thresh & abs(peaks$res.72.geno.FC) > fcthresh) | 
    (peaks$res.wt.time.padj < thresh & abs(peaks$res.wt.time.FC) > fcthresh) | (peaks$res.mut.time.padj < 
    thresh & abs(peaks$res.mut.time.FC) > fcthresh))]
```

Then, on the basis of the DESeq2 analysis, we can define different classes of peaks and use statistical outputs to identify them. In the following, ‘uniform’ is meant to indicate a genotype-independent state, whereas genotype-dependent states (early accessibility, late repression, late accessibility) are preceded by whether the effect is observed in the wild-type or the mutant class. Below, we plot what these groups look like along with their names in case that is a more intuitive presentation of the classes. Note that the way I define the classes in the next chunk is not necessarly exclusive (one peak can end up in more than one group). I filter these based on visual examination of the peak classes in a subsequent chunk.

```
# uniform early groups

unif.early.mut.rep = dynamic[dynamic$res.72.geno.padj < thresh & dynamic$res.72.geno.FC < 
    -1 * fcthresh & dynamic$res.mut.time.padj < thresh & dynamic$res.mut.time.FC < 
    -1 * fcthresh]

# the second two of these have pattern-dependent 'repression' or loss of
# accessibility.

unif.early.wt.rep = dynamic[dynamic$res.72.geno.padj < thresh & dynamic$res.72.geno.FC > 
    fcthresh & dynamic$res.wt.time.padj < thresh & dynamic$res.wt.time.FC < -1 * 
    fcthresh]

unif.early.unif.rep = dynamic[dynamic$res.wt.time.padj < thresh & dynamic$res.wt.time.FC < 
    -1 * fcthresh & dynamic$res.mut.time.padj < thresh & dynamic$res.mut.time.FC < 
    -1 * fcthresh]

# pattern dependent early groups, 'constitutive' distinguishes classes where a
# region opens up and remains open in the late timepoint.

wt.early.wt.rep = dynamic[dynamic$res.12.geno.padj < thresh & dynamic$res.12.geno.FC < 
    -1 * fcthresh & dynamic$res.wt.time.padj < thresh & dynamic$res.wt.time.FC < 
    -1 * fcthresh]

mut.early.mut.rep = dynamic[dynamic$res.12.geno.padj < thresh & dynamic$res.12.geno.FC > 
    fcthresh & dynamic$res.mut.time.padj < thresh & dynamic$res.mut.time.FC < -1 * 
    fcthresh]

wt.early.constitutive = dynamic[dynamic$res.12.geno.padj < thresh & dynamic$res.12.geno.FC < 
    -1 * fcthresh & dynamic$res.72.geno.padj < thresh & dynamic$res.72.geno.FC < 
    -1 * fcthresh]

mut.early.constitutive = dynamic[dynamic$res.12.geno.padj < thresh & dynamic$res.12.geno.FC > 
    fcthresh & dynamic$res.72.geno.padj < thresh & dynamic$res.72.geno.FC > fcthresh]

# late groups

unif.late = dynamic[dynamic$res.wt.time.padj < thresh & dynamic$res.wt.time.FC > 
    fcthresh & dynamic$res.mut.time.padj < thresh & dynamic$res.mut.time.FC > fcthresh]

wt.late = dynamic[dynamic$res.72.geno.padj < thresh & dynamic$res.72.geno.FC < -1 * 
    fcthresh & dynamic$res.wt.time.padj < thresh & dynamic$res.wt.time.FC > fcthresh]

mut.late = dynamic[dynamic$res.72.geno.padj < thresh & dynamic$res.72.geno.FC > fcthresh & 
    dynamic$res.mut.time.padj < thresh & dynamic$res.mut.time.FC > fcthresh]
```

This gets all of the conceptually sound groups, and I check this assumption later on. Let’s calculate at the moment how many of the dynamic peaks we can account for.

```
l = c(length(unif.early.wt.rep), length(unif.early.mut.rep), length(unif.early.unif.rep), 
    length(wt.early.wt.rep), length(mut.early.mut.rep), length(wt.early.constitutive), 
    length(mut.early.constitutive), length(unif.late), length(wt.late), length(mut.late))

names(l) = c("unif.early.wt.rep", "unif.early.mut.rep", "unif.early.unif.rep", "wt.early.wt.rep", 
    "mut.early.mut.rep", "wt.early.constitutive", "mut.early.constitutive", "unif.late", 
    "wt.late", "mut.late")

l
```

```
##      unif.early.wt.rep     unif.early.mut.rep    unif.early.unif.rep 
##                     75                    333                    948 
##        wt.early.wt.rep      mut.early.mut.rep  wt.early.constitutive 
##                     32                    102                     83 
## mut.early.constitutive              unif.late                wt.late 
##                     77                    780                    322 
##               mut.late 
##                    399
```

Again, these aren’t the final numbers of peaks per class.

The chunk below creates logical vectors that reflect the categorization of peaks that are compatible with adding back to the original peaks list.

```
unif.early.unif.rep.l = names(peaks) %in% names(unif.early.unif.rep)
unif.early.wt.rep.l = names(peaks) %in% names(unif.early.wt.rep)
unif.early.mut.rep.l = names(peaks) %in% names(unif.early.mut.rep)
wt.early.wt.rep.l = names(peaks) %in% names(wt.early.wt.rep)
mut.early.mut.rep.l = names(peaks) %in% names(mut.early.mut.rep)
wt.early.constitutive.l = names(peaks) %in% names(wt.early.constitutive)
mut.early.constitutive.l = names(peaks) %in% names(mut.early.constitutive)
unif.late.l = names(peaks) %in% names(unif.late)
wt.late.l = names(peaks) %in% names(wt.late)
mut.late.l = names(peaks) %in% names(mut.late)

# the following lines sort out 'double hits' that were not filtered for
# explicitly in the original designation of classes. This is an all-or-nothing
# filtering, and could possibly be more sophisticated by using DESeq2 outputs not
# previously considered to break ties.

unif.early.mut.rep.l[unif.early.unif.rep.l] = FALSE
unif.early.wt.rep.l[unif.early.unif.rep.l] = FALSE
unif.early.unif.rep.l[wt.early.wt.rep.l] = FALSE
unif.early.unif.rep.l[mut.early.mut.rep.l] = FALSE
unif.early.unif.rep.l[wt.early.constitutive.l] = FALSE
unif.early.unif.rep.l[mut.early.constitutive.l] = FALSE
unif.early.wt.rep.l[mut.early.constitutive.l] = FALSE
unif.early.mut.rep.l[wt.early.constitutive.l] = FALSE
unif.early.mut.rep.l[wt.late.l] = FALSE
unif.early.mut.rep.l[mut.early.mut.rep.l] = FALSE
wt.early.constitutive.l[wt.early.wt.rep.l] = FALSE
wt.early.constitutive.l[wt.late.l] = FALSE
mut.early.constitutive.l[mut.early.mut.rep.l] = FALSE
mut.early.constitutive.l[mut.late.l] = FALSE
unif.late.l[mut.early.constitutive.l] = FALSE
unif.late.l[wt.late.l] = FALSE
unif.late.l[mut.late.l] = FALSE

# the following lines assemble this data into a concise table.

class.table = data.frame(unif.early.unif.rep = unif.early.unif.rep.l, unif.early.wt.rep = unif.early.wt.rep.l, 
    unif.early.mut.rep = unif.early.mut.rep.l, wt.early.late.rep = wt.early.wt.rep.l, 
    mut.early.late.rep = mut.early.mut.rep.l, wt.early.constitutive = wt.early.constitutive.l, 
    mut.early.constitutive = mut.early.constitutive.l, unif.late = unif.late.l, wt.late = wt.late.l, 
    mut.late = mut.late.l)

colSums(class.table)
```

```
##    unif.early.unif.rep      unif.early.wt.rep     unif.early.mut.rep 
##                    910                     51                    256 
##      wt.early.late.rep     mut.early.late.rep  wt.early.constitutive 
##                     32                    102                     68 
## mut.early.constitutive              unif.late                wt.late 
##                     52                    725                    322 
##               mut.late 
##                    399
```

We can calculate what fraction of the original general ‘dynamic’ class is captured within this set of stringently defined classes.

```
cat(paste0("percent captured\n", round(sum(colSums(class.table))/length(dynamic) * 
    100, 2), "%"))
```

```
## percent captured
## 43.06%
```

This isn’t 100%, and below I demonstrate why that is.

First, let’s plot the scaled accessibility values per class.

These look good, we can append these logical classifiers to the peak table.

```
meta = mcols(peaks)
mcols(peaks) = NULL
newmeta = cbind(meta, class.table)
mcols(peaks) = newmeta
```

### Did we miss anything?

One prediction is that our inability to account for 100% of the peaks that were originally called ‘dynamic’ is that to designate the ‘dynamic’ class, I asked for any peak that had at least one statistically significant difference with an absolute magnitude of 2-fold. All of these classes that I designated were, by definition, those with two statistically significant differences > 2 fold. We would expect a significant number of the non-captured ‘dynamic’ regions to have only one statistically significant difference.

```
in.class = peaks[peaks$unif.early.unif.rep | peaks$unif.early.wt.rep | peaks$unif.early.mut.rep | 
    peaks$wt.early.late.rep | peaks$mut.early.late.rep | peaks$wt.early.constitutive | 
    peaks$mut.early.constitutive | peaks$unif.late | peaks$wt.late | peaks$mut.late]

dynamic.in.classes = dynamic[names(dynamic) %in% names(in.class)]
dynamic.no.classes = dynamic[!names(dynamic) %in% names(in.class)]

# we can calculate the number of above threshold significant tests/fold changes
# in each group using the following:

dynamic.in.class.sigs = cbind(dynamic.in.classes$res.wt.time.padj < thresh & abs(dynamic.in.classes$res.wt.time.FC) > 
    fcthresh, dynamic.in.classes$res.mut.time.padj < thresh & abs(dynamic.in.classes$res.mut.time.FC) > 
    fcthresh, dynamic.in.classes$res.12.geno.padj < thresh & abs(dynamic.in.classes$res.12.geno.FC) > 
    fcthresh, dynamic.in.classes$res.72.geno.padj < thresh & abs(dynamic.in.classes$res.72.geno.FC) > 
    fcthresh)

dynamic.no.class.sigs = cbind(dynamic.no.classes$res.wt.time.padj < thresh & abs(dynamic.no.classes$res.wt.time.FC) > 
    fcthresh, dynamic.no.classes$res.mut.time.padj < thresh & abs(dynamic.no.classes$res.mut.time.FC) > 
    fcthresh, dynamic.no.classes$res.12.geno.padj < thresh & abs(dynamic.no.classes$res.12.geno.FC) > 
    fcthresh, dynamic.no.classes$res.72.geno.padj < thresh & abs(dynamic.no.classes$res.72.geno.FC) > 
    fcthresh)
```

Then, if we calculate the number of above-threshold successful tests, they should all be greater than one for the peaks that were assigned to a class.

```
all(rowSums(dynamic.in.class.sigs) > 1)
```

```
## [1] TRUE
```

```
table(rowSums(dynamic.in.class.sigs))
```

```
## 
##    2    3    4 
## 2709  191   17
```

And we predict that most of the non-classified ‘dynamic’ regions are only above threshold in one test.

```
table(rowSums(dynamic.no.class.sigs))
```

```
## 
##    1    2 
## 3809   49
```

Which is true. There are 49 regions that could represent a true ‘missed’ class.

```
maybe.missed = dynamic.no.class.sigs[rowSums(dynamic.no.class.sigs) == 2, ]

colSums(maybe.missed)
```

```
## [1] 13 36 49  0
```

Every remaining double positive is significantly enriched for differences between genotypes in the early timepoint.

Most of these also have significant differences in the mutant over time. Visual examination of these indicates that they represent weak classes of late-accessible sites where the fold change in accessibility over time is only significant in one genotype and not the other. I am ok with missing these 49 regions.

### Making summary plots of the DESeq2 analysis

We can do PCA to summarize the major sources of variance in these data. Below, I limit the PCA to all of the classified peaks. It should be noted that essentially the same result is obtained by performing PCA on the entire dataset (all peaks, both significantly different as well as invariant.)

```
newdyn = as.logical(apply(class.table, 1, sum))

# principal component analysis:
rld2 = rlog(dds[newdyn], blind = FALSE)
pca = plotPCA(rld2, intgroup = c("genotype", "time"), returnData = TRUE, ntop = length(rld2))
percentVar = round(100 * attr(pca, "percentVar"))

# dev.new(width = 6, height = 6)
ggplot(pca, aes(PC1, PC2, color = genotype, shape = time)) + geom_point(size = 3) + 
    xlab(paste0("PC1: ", percentVar[1], "% variance")) + ylab(paste0("PC2: ", percentVar[2], 
    "% variance")) + scale_color_manual(values = c("tomato", "blue")) + theme(axis.line = element_line(color = "black")) + 
    theme_bw() + xlim(-55, 55) + ylim(-40, 40) + coord_fixed()
```

We can plot the ‘volcano’ plots that capture the results of the statistical tests from DESeq2. We can scale the point size to reflect the mean ATAC signal for each component of the plot.

```
volcanize = function(fc, padj, meanSig, point.amplifier = 1, alpha.range = 0.25, 
    xlims = c(-6, 6), ylims = c(0, 70), title, plot.legend = TRUE, xlab.add.on = "") {
    plot.range = log2(meanSig + 1)/max(log2(meanSig + 1))
    plot.range = 4 * plot.range^2
    plot(fc, -log10(padj), cex = point.amplifier * plot.range, col = rgb(0, 0, 0, 
        alpha.range), pch = 20, xlim = xlims, ylim = ylims, bty = "n", ylab = "-log10 adjusted p-value", 
        xlab = paste0("log2 Fold Change", xlab.add.on), las = 1, main = title)
    abline(h = -log10(thresh), col = "red", lty = 3)
    abline(v = c((-1 * fcthresh), fcthresh), col = "red", lty = 3)
    if (plot.legend) {
        legend.dots = quantile(point.amplifier * plot.range)
        legend("topright", legend = c("0", "0.25", "0.5", "0.75", "1.0"), col = rgb(0, 
            0, 0, quantile(alpha.range)), pch = 20, pt.cex = c(0, 1, 2, 3, 4), title = "ATAC signal", 
            cex = 0.8)
    }
}
```

```
volcanize(fc = peaks$res.12.geno.FC, padj = peaks$res.12.geno.padj, meanSig = peaks$baseMean, 
    title = "Between Genotypes, NC14+12")
```

```
volcanize(fc = peaks$res.72.geno.FC, padj = peaks$res.72.geno.padj, meanSig = peaks$baseMean, 
    title = "Between Genotypes, NC14+72")
```

```
volcanize(fc = peaks$res.wt.time.FC, padj = peaks$res.wt.time.padj, meanSig = peaks$baseMean, 
    title = "Between Timepoints, wild-type")
```

```
volcanize(fc = peaks$res.mut.time.FC, padj = peaks$res.mut.time.padj, meanSig = peaks$baseMean, 
    title = "Between Timepoints, mutant")
```

```
# export some publication quality images.

png(filename = "~/Dropbox/Blythe Lab/Patternless Paper/Code For Figures/Volcano1.png", 
    width = 6, height = 6, units = "in", res = 300)
volcanize(fc = peaks$res.12.geno.FC, padj = peaks$res.12.geno.padj, meanSig = peaks$baseMean, 
    title = "Between Genotypes, NC14+12", xlab.add.on = " (mut/wt)")
dev.off()

png(filename = "~/Dropbox/Blythe Lab/Patternless Paper/Code For Figures/Volcano2.png", 
    width = 6, height = 6, units = "in", res = 300)
volcanize(fc = peaks$res.72.geno.FC, padj = peaks$res.72.geno.padj, meanSig = peaks$baseMean, 
    title = "Between Genotypes, NC14+72", plot.legend = FALSE, xlab.add.on = " (mut/wt)")
dev.off()

png(filename = "~/Dropbox/Blythe Lab/Patternless Paper/Code For Figures/Volcano3.png", 
    width = 6, height = 6, units = "in", res = 300)
volcanize(fc = peaks$res.wt.time.FC, padj = peaks$res.wt.time.padj, meanSig = peaks$baseMean, 
    title = "Between Timepoints, wild-type", plot.legend = FALSE, xlab.add.on = " (late/early)")
dev.off()

png(filename = "~/Dropbox/Blythe Lab/Patternless Paper/Code For Figures/Volcano4.png", 
    width = 6, height = 6, units = "in", res = 300)
volcanize(fc = peaks$res.mut.time.FC, padj = peaks$res.mut.time.padj, meanSig = peaks$baseMean, 
    title = "Between Timepoints, mutant", plot.legend = FALSE, xlab.add.on = " (late/early)")
dev.off()
```

### Observations

The PCA result suggests that the primary driver of ‘differences’ between samples is developmental time (PC1 separates timepoints). 66% of the variance is captured by developmental time. The second driver is maternal patterning systems, (PC2 separates genotypes). 21% of the variance is captured by genotype. The early timepoints are more similar to one another than the later ones. This is consistent with prior observations about the virtual homogeneity of early developmental states.

The classes we generate indicate that a substantial amount of changes in chromatin accessibility occur uniformly (genotype independent) and that there are roughly a similar number of gains and losses in chromatin accessibility for regions that change uniformly.

There are major sources of gastrula stage repression which may be common, or may be patterning dependent. For instance, ‘uniform early uniform rep’ shows gastrula stage repression of 910 regions. The assumed ‘repressive’ activity for ‘mut/wt early, late rep’ could be similar, or could be a unique source. However, for the “uniform early mut/wt rep” classes, something distinguishes these regions from one another. This could either be maintained accessibility (anti-repression) in one genotype, or genotype-specific repression.

There are major sources of late chromatin accessibility that stem either from uniform or pattern-dependent sources. Why these regions open late is an open question, either the unavailability of an activator/pioneer, or the early repressive activity of some unknown factor (or a combination of the two).

To get at these a bit more, we can investigate whether there are any enriched motifs within these regions.

## Outputting sequences for MEME analysis

Meme wants regions that are no larger than 100 bp as input for analysis. To get these sequences, we can get the peaks per class, center over the maximum peak accessibility position, and resize to 100 bp, then spit out the sequences to a fasta formatted file.

```
# I've set this to not evaluate since I don't want to overwrite what I've done in
# the true analysis. Please remove the 'eval = FALSE' above if you want to run
# this yourself.

# we want a set of non-dynamic peaks to use as a background set for each
# comparison
bgpeaks = peaks[!peaks %in% dynamic]

set.seed(138)

bgset = sample(bgpeaks, size = 10000)
start(bgset) = bgset$max
end(bgset) = bgset$max
bgset = resize(bgset, width = 100, fix = "center")
bgseqs = getSeq(Dmelanogaster, bgset)
export(bgseqs, con = paste0(sequence.output.directory, "/non-dynamic_background.txt"), 
    format = "fasta")

get.my.seqs = function(subset, p = peaks, dir = sequence.output.directory) {
    regions = p[mcols(p)[subset][, 1]]
    start(regions) = regions$max
    end(regions) = regions$max
    regions = resize(regions, width = 100, fix = "center")
    regionseqs = getSeq(Dmelanogaster, regions)
    export(regionseqs, con = paste0(dir, "/", subset, ".txt"), format = "fasta")
}
get.my.seqs(subset = "unif.early.unif.rep")
get.my.seqs(subset = "unif.early.wt.rep")
get.my.seqs(subset = "unif.early.mut.rep")
get.my.seqs(subset = "wt.early.late.rep")
get.my.seqs(subset = "mut.early.late.rep")
get.my.seqs(subset = "wt.early.constitutive")
get.my.seqs(subset = "mut.early.constitutive")
get.my.seqs(subset = "unif.late")
get.my.seqs(subset = "wt.late")
get.my.seqs(subset = "mut.late")
```

MEME-ChIP analysis is run on the output files using discriminative mode, limiting to “Fly DNA” and searching against combined Drosophila databases. The background model is ‘1st order model of sequences’, and we search for up to 5 motifs. Otherwise, everything is default.

http://meme-suite.org/tools/meme-chip

The meme analysis can be done from the command line (but this markdown is set to not reproduce this every time since it is time-consuming.)

```
export PATH=$HOME/meme/bin:$HOME/meme/libexec/meme-5.1.0:$PATH
bgfile=~/Dropbox/HTseq/Analysis/Patternless_ATAC/Peak_Class_Sequences_191031/non-dynamic_background.txt
dbpath=/Applications/meme/motif_databases/FLY

seqfile=~/Dropbox/HTseq/Analysis/Patternless_ATAC/Peak_Class_Sequences_191031/unif.late.txt

meme-chip -oc ~/Dropbox/HTseq/Analysis/Patternless_ATAC/Peak_Class_Sequences_191031/meme.unif.late -neg $bgfile -order 1 -psp-gen -db $dbpath/fly_factor_survey.meme -db $dbpath/dmmpmm2009.meme -db $dbpath/flyreg.v2.meme -db $dbpath/idmmpmm2009.meme -db $dbpath/OnTheFly_2014_Drosophila.meme -meme-mod zoops -meme-maxw 30 -meme-nmotifs 5 -meme-searchsize 100000 -dreme-e 0.05 -centrimo-score 5.0 -centrimo-ethresh 10.0 $seqfile

seqfile=~/Dropbox/HTseq/Analysis/Patternless_ATAC/Peak_Class_Sequences_191031/wt.late.txt

meme-chip -oc ~/Dropbox/HTseq/Analysis/Patternless_ATAC/Peak_Class_Sequences_191031/meme.wt.late -neg $bgfile -order 1 -psp-gen -db $dbpath/fly_factor_survey.meme -db $dbpath/dmmpmm2009.meme -db $dbpath/flyreg.v2.meme -db $dbpath/idmmpmm2009.meme -db $dbpath/OnTheFly_2014_Drosophila.meme -meme-mod zoops -meme-maxw 30 -meme-nmotifs 5 -meme-searchsize 100000 -dreme-e 0.05 -centrimo-score 5.0 -centrimo-ethresh 10.0 $seqfile

seqfile=~/Dropbox/HTseq/Analysis/Patternless_ATAC/Peak_Class_Sequences_191031/mut.late.txt

meme-chip -oc ~/Dropbox/HTseq/Analysis/Patternless_ATAC/Peak_Class_Sequences_191031/meme.mut.late -neg $bgfile -order 1 -psp-gen -db $dbpath/fly_factor_survey.meme -db $dbpath/dmmpmm2009.meme -db $dbpath/flyreg.v2.meme -db $dbpath/idmmpmm2009.meme -db $dbpath/OnTheFly_2014_Drosophila.meme -meme-mod zoops -meme-maxw 30 -meme-nmotifs 5 -meme-searchsize 100000 -dreme-e 0.05 -centrimo-score 5.0 -centrimo-ethresh 10.0 $seqfile

seqfile=~/Dropbox/HTseq/Analysis/Patternless_ATAC/Peak_Class_Sequences_191031/unif.early.unif.rep.txt

meme-chip -oc ~/Dropbox/HTseq/Analysis/Patternless_ATAC/Peak_Class_Sequences_191031/meme.unif.early.unif.rep -neg $bgfile -order 1 -psp-gen -db $dbpath/fly_factor_survey.meme -db $dbpath/dmmpmm2009.meme -db $dbpath/flyreg.v2.meme -db $dbpath/idmmpmm2009.meme -db $dbpath/OnTheFly_2014_Drosophila.meme -meme-mod zoops -meme-maxw 30 -meme-nmotifs 5 -meme-searchsize 100000 -dreme-e 0.05 -centrimo-score 5.0 -centrimo-ethresh 10.0 $seqfile

seqfile=~/Dropbox/HTseq/Analysis/Patternless_ATAC/Peak_Class_Sequences_191031/unif.early.wt.rep.txt

meme-chip -oc ~/Dropbox/HTseq/Analysis/Patternless_ATAC/Peak_Class_Sequences_191031/meme.unif.early.wt.rep -neg $bgfile -order 1 -psp-gen -db $dbpath/fly_factor_survey.meme -db $dbpath/dmmpmm2009.meme -db $dbpath/flyreg.v2.meme -db $dbpath/idmmpmm2009.meme -db $dbpath/OnTheFly_2014_Drosophila.meme -meme-mod zoops -meme-maxw 30 -meme-nmotifs 5 -meme-searchsize 100000 -dreme-e 0.05 -centrimo-score 5.0 -centrimo-ethresh 10.0 $seqfile

seqfile=~/Dropbox/HTseq/Analysis/Patternless_ATAC/Peak_Class_Sequences_191031/unif.early.mut.rep.txt

meme-chip -oc ~/Dropbox/HTseq/Analysis/Patternless_ATAC/Peak_Class_Sequences_191031/meme.unif.early.mut.rep -neg $bgfile -order 1 -psp-gen -db $dbpath/fly_factor_survey.meme -db $dbpath/dmmpmm2009.meme -db $dbpath/flyreg.v2.meme -db $dbpath/idmmpmm2009.meme -db $dbpath/OnTheFly_2014_Drosophila.meme -meme-mod zoops -meme-maxw 30 -meme-nmotifs 5 -meme-searchsize 100000 -dreme-e 0.05 -centrimo-score 5.0 -centrimo-ethresh 10.0 $seqfile

seqfile=~/Dropbox/HTseq/Analysis/Patternless_ATAC/Peak_Class_Sequences_191031/wt.early.late.rep.txt

meme-chip -oc ~/Dropbox/HTseq/Analysis/Patternless_ATAC/Peak_Class_Sequences_191031/meme.wt.early.late.rep -neg $bgfile -order 1 -psp-gen -db $dbpath/fly_factor_survey.meme -db $dbpath/dmmpmm2009.meme -db $dbpath/flyreg.v2.meme -db $dbpath/idmmpmm2009.meme -db $dbpath/OnTheFly_2014_Drosophila.meme -meme-mod zoops -meme-maxw 30 -meme-nmotifs 5 -meme-searchsize 100000 -dreme-e 0.05 -centrimo-score 5.0 -centrimo-ethresh 10.0 $seqfile

seqfile=~/Dropbox/HTseq/Analysis/Patternless_ATAC/Peak_Class_Sequences_191031/mut.early.late.rep.txt

meme-chip -oc ~/Dropbox/HTseq/Analysis/Patternless_ATAC/Peak_Class_Sequences_191031/meme.mut.early.late.rep -neg $bgfile -order 1 -psp-gen -db $dbpath/fly_factor_survey.meme -db $dbpath/dmmpmm2009.meme -db $dbpath/flyreg.v2.meme -db $dbpath/idmmpmm2009.meme -db $dbpath/OnTheFly_2014_Drosophila.meme -meme-mod zoops -meme-maxw 30 -meme-nmotifs 5 -meme-searchsize 100000 -dreme-e 0.05 -centrimo-score 5.0 -centrimo-ethresh 10.0 $seqfile

seqfile=~/Dropbox/HTseq/Analysis/Patternless_ATAC/Peak_Class_Sequences_191031/wt.early.constitutive.txt

meme-chip -oc ~/Dropbox/HTseq/Analysis/Patternless_ATAC/Peak_Class_Sequences_191031/meme.wt.early.constitutive -neg $bgfile -order 1 -psp-gen -db $dbpath/fly_factor_survey.meme -db $dbpath/dmmpmm2009.meme -db $dbpath/flyreg.v2.meme -db $dbpath/idmmpmm2009.meme -db $dbpath/OnTheFly_2014_Drosophila.meme -meme-mod zoops -meme-maxw 30 -meme-nmotifs 5 -meme-searchsize 100000 -dreme-e 0.05 -centrimo-score 5.0 -centrimo-ethresh 10.0 $seqfile

seqfile=~/Dropbox/HTseq/Analysis/Patternless_ATAC/Peak_Class_Sequences_191031/mut.early.constitutive.txt

meme-chip -oc ~/Dropbox/HTseq/Analysis/Patternless_ATAC/Peak_Class_Sequences_191031/meme.mut.early.constitutive -neg $bgfile -order 1 -psp-gen -db $dbpath/fly_factor_survey.meme -db $dbpath/dmmpmm2009.meme -db $dbpath/flyreg.v2.meme -db $dbpath/idmmpmm2009.meme -db $dbpath/OnTheFly_2014_Drosophila.meme -meme-mod zoops -meme-maxw 30 -meme-nmotifs 5 -meme-searchsize 100000 -dreme-e 0.05 -centrimo-score 5.0 -centrimo-ethresh 10.0 $seqfile
```

## Plotting a representative example for each group.

We want to show a nice example of a peak from each group. While selecting an example is admittedly a subjective exercise, I use the following loose rules.

- Pick a region that is near a gene that people know things about.
- Try to limit regions of interest to those that have relatively high ‘mean accessibility’. This parameter is summarized in the ‘baseMean’ column in the peak table.

```
ROI.unif.early.unif.rep = "chr2L:6372869-6374281"  #slam
ROI.unif.early.wt.rep = "chrX:15646660-15647544"  #sog shadow
ROI.unif.early.mut.rep = "chr2L:3614548-3615690"  #odd-10
ROI.wt.early.late.rep = "chrX:2438209-2438868"  # gt-10 (many gap gene enhancers are in this group)
ROI.mut.early.late.rep = "chr2L:20770258-20770894"  #cad (also get tll and hkb, byn, and cad)
ROI.wt.early.constitutive = "chr3R:16700717-16701503"  #Ubx
ROI.mut.early.constitutive = "chr3R:28588461-28589156"  #fkh
ROI.unif.late = "chr2L:523107-523883"  #ush
ROI.wt.late = "chr2L:3817914-3818519"  #slp1
ROI.mut.late = "chr2R:20091920-20092449"  #18w

ROI.bonus.wingless = "chr2L:7305625-7306841"

load(openwigfile)

# we impose some smoothing to the data for presentation purposes.
bc = lapply(bc, function(x) {
    # cat('*')
    a = score(x)
    b = rollmean(as.zoo(a), 10)
    c = rep(0, length(a))
    c[index(b)] = b
    score(x) = c
    return(x)
})
```

```
names(bc) = c("WT NC14+12", "WT NC14+72", "mut NC14+12", "mut NC14+72")

gvizzer = function(ROI, view.width = 10000, counts = bc, ylims = c(0, 20), title, 
    shifter = 0, highlight = TRUE) {
    chr = substring(ROI, 1, (regexpr(":", ROI) - 1))
    ofrom = as.numeric(substring(ROI, (regexpr(":", ROI) + 1), (regexpr("-", ROI) - 
        1)))
    oto = as.numeric(substring(ROI, (regexpr("-", ROI) + 1), nchar(ROI)))
    
    oregion = GRanges(chr, IRanges(ofrom, oto))
    region = resize(oregion, width = view.width, fix = "center")
    start(region) = start(region) + shifter
    end(region) = end(region) + shifter
    
    from = as.numeric(start(region))
    to = as.numeric(end(region))
    
    title = paste0(title, ": ", ROI)
    
    grtrack = GeneRegionTrack(TxDb.Dmelanogaster.UCSC.dm6.ensGene, genome = "dm6", 
        from = from, to = to, chromosome = chr, stacking = "dense")
    gtr = GenomeAxisTrack(scale = 1000)
    
    trackcols = c("midnightblue", "midnightblue", "brown", "brown")
    
    datatracks = sapply(c(1:4), function(x) DataTrack(counts[[x]], name = names(counts[x]), 
        type = "polygon", col.mountain = trackcols[x], fill.mountain = rep(trackcols[x], 
            2)))
    
    if (highlight) {
        htrack = HighlightTrack(trackList = list(datatracks[[1]], datatracks[[2]], 
            datatracks[[3]], datatracks[[4]], grtrack), range = oregion)
        plotTracks(trackList = list(gtr, htrack), from = from, to = to, chromosome = chr, 
            main = title, ylim = ylims, cex.main = 1)
    } else {
        plotTracks(trackList = list(gtr, datatracks[[1]], datatracks[[2]], datatracks[[3]], 
            datatracks[[4]], grtrack), from = from, to = to, chromosome = chr, main = title, 
            ylim = ylims, cex.main = 1)
    }
    
    
}
```

```
gvizzer(ROI.unif.early.unif.rep, view.width = 25000, title = "slam", shifter = 5000)
```

```
png(filename = "~/Dropbox/Blythe Lab/Patternless Paper/Code For Figures/slam_unif_early_unif_rep_example.GViz.png", 
    width = 2400, height = 1500, units = "px", res = 300)
gvizzer(ROI.unif.early.unif.rep, view.width = 25000, title = "slam", shifter = 5000)
dev.off()
```

```
gvizzer(ROI.unif.early.wt.rep, view.width = 50000, ylims = c(0, 15), title = "sog shadow", 
    shifter = -10000)
```

```
gvizzer(ROI.unif.early.mut.rep, view.width = 30000, ylims = c(0, 15), title = "odd -10", 
    shifter = -5000)
```

```
gvizzer(ROI.wt.early.late.rep, view.width = 25000, ylims = c(0, 10), title = "gt -10", 
    shifter = 0)
```

```
png(filename = "~/Dropbox/Blythe Lab/Patternless Paper/Code For Figures/gt10_wt_early_late_rep_example.GViz.png", 
    width = 2400, height = 1500, units = "px", res = 300)
gvizzer(ROI.wt.early.late.rep, view.width = 25000, ylims = c(0, 10), title = "gt -10", 
    shifter = 0)
dev.off()
```

```
gvizzer(ROI.mut.early.late.rep, view.width = 27000, ylims = c(0, 20), title = "cad", 
    shifter = 2400)
```

```
gvizzer(ROI.wt.early.constitutive, view.width = 40000, ylims = c(0, 15), title = "Ubx", 
    shifter = 5000)
```

```
gvizzer(ROI.mut.early.constitutive, view.width = 25000, ylims = c(0, 15), title = "fkh", 
    shifter = -5000)
```

```
gvizzer(ROI.unif.late, view.width = 25000, ylims = c(0, 15), title = "ush")
```

```
gvizzer(ROI.wt.late, view.width = 25000, ylims = c(0, 15), title = "slp1 '5'", shifter = 5000)
```

```
png(filename = "~/Dropbox/Blythe Lab/Patternless Paper/Code For Figures/slp5_wt.late_class_example.GViz.png", 
    width = 2400, height = 1500, units = "px", res = 300)
gvizzer(ROI.wt.late, view.width = 25000, ylims = c(0, 18), title = "slp1 '5'", shifter = 8000)
dev.off()
```

```
gvizzer(ROI.mut.late, view.width = 50000, ylims = c(0, 15), title = "18w", shifter = 4000)
```

A bonus region (wingless)

```
gvizzer(ROI.bonus.wingless, view.width = 50000, ylims = c(0, 18), title = "wg", shifter = 6000, 
    highlight = FALSE)
```

```
png(filename = "~/Dropbox/Blythe Lab/Patternless Paper/Code For Figures/wingless_example.GViz.png", 
    width = 2400, height = 1500, units = "px", res = 300)
gvizzer(ROI.bonus.wingless, view.width = 50000, ylims = c(0, 18), title = "wg", shifter = 6000, 
    highlight = FALSE)
dev.off()
```

## How sensitive are different peak classes to loss of Bcd or Zld?

We find through the meme analysis that two classes, `wt.early.late.rep` and `wt.early.constitutive` are enriched for both Zld and Bcd motifs. Zld alone is also enriched within `unif.early.unif.rep` and `unif.early.mut.rep`. We have previously shown that Bcd has ‘pioneer-like’ activity and can drive accessibility at a subset of its sites. Zld is a pioneer. Let’s load up our previous Bcd and Zld ATAC experiments, generate DESeq2 analysis to this set of peaks, and evaluate whether Zld- or Bcd- dependence is enriched within any of the newly designated peak classes overall. Going into this, we hypothesize that Zld will have a broad effect on many of the early-accessible regions, and that Bcd will have an effect on the two classes where we see enrichment of sites.

Note, the prior Zld and Bcd ATAC was performed under similar conditions, but only considered the NC14+12 timepoint, so this limits some of the comparisons we can make.

The DESeq analysis will be performed as above, independently on the previously published datasets, with appropriate changes made to the results table as necessary.

```
## [1] "Intercept"          "genotype_bcd_vs_wt" "genotype_zld_vs_wt"
```

We can make volcano plots for each of these genotypes:

```
volcanize(fc = htable$res.bcd.FC, padj = htable$res.bcd.padj, meanSig = htable$baseMean, 
    title = "bcd, NC14+12", xlab.add.on = " (bcd/wt)")
```

```
volcanize(fc = htable$res.zld.FC, padj = htable$res.zld.padj, meanSig = htable$baseMean, 
    title = "zld, NC14+12", xlab.add.on = " (zld/wt)")
```

Zld clearly has an effect on the system.

To get a sense of whether the set of zld-sensitive or bcd-sensitive sites are enriched for any peak class, we can perform fisher’s exact tests on the class x bcd/zld categorizations.

```
fishing = function(class1, class2, class1Name, class2Name) {
    contingency_table = rbind(c(sum(class1 & class2), sum(class1 & !class2)), c(sum(!class1 & 
        class2), sum(!class1 & !class2)))
    rownames(contingency_table) = c(class1Name, paste0("!", class1Name))
    colnames(contingency_table) = c(class2Name, paste0("!", class2Name))
    ft = fisher.test(contingency_table)
    list(fisher = ft, contingency = contingency_table)
}
```

```
fishing(class1 = (htable$res.bcd.padj < thresh & htable$res.bcd.FC < -1 * fcthresh), 
    class2 = peaks$wt.early.late.rep, class1Name = "bcd.down", class2Name = "wt.early.late.rep")
```

```
## $fisher
## 
##  Fisher's Exact Test for Count Data
## 
## data:  contingency_table
## p-value < 2.2e-16
## alternative hypothesis: true odds ratio is not equal to 1
## 95 percent confidence interval:
##  154.0107 762.5557
## sample estimates:
## odds ratio 
##   340.4398 
## 
## 
## $contingency
##           wt.early.late.rep !wt.early.late.rep
## bcd.down                 19                113
## !bcd.down                13              26183
```

```
fishing(class1 = (htable$res.zld.padj < thresh & htable$res.zld.FC < -1 * fcthresh), 
    class2 = peaks$wt.early.late.rep, class1Name = "zld.down", class2Name = "wt.early.late.rep")
```

```
## $fisher
## 
##  Fisher's Exact Test for Count Data
## 
## data:  contingency_table
## p-value = 9.669e-16
## alternative hypothesis: true odds ratio is not equal to 1
## 95 percent confidence interval:
##   9.455909 49.399065
## sample estimates:
## odds ratio 
##    20.8429 
## 
## 
## $contingency
##           wt.early.late.rep !wt.early.late.rep
## zld.down                 22               2510
## !zld.down                10              23786
```

```
fishing(class1 = (htable$res.bcd.padj < thresh & htable$res.bcd.FC < -1 * fcthresh), 
    class2 = peaks$mut.early.late.rep, class1Name = "bcd.down", class2Name = "mut.early.late.rep")
```

```
## $fisher
## 
##  Fisher's Exact Test for Count Data
## 
## data:  contingency_table
## p-value = 0.4017
## alternative hypothesis: true odds ratio is not equal to 1
## 95 percent confidence interval:
##   0.04908 11.43655
## sample estimates:
## odds ratio 
##    1.97219 
## 
## 
## $contingency
##           mut.early.late.rep !mut.early.late.rep
## bcd.down                   1                 131
## !bcd.down                101               26095
```

```
fishing(class1 = (htable$res.zld.padj < thresh & htable$res.zld.FC < -1 * fcthresh), 
    class2 = peaks$unif.early.unif.rep, class1Name = "zld.down", class2Name = "unif.early.unif.rep")
```

```
## $fisher
## 
##  Fisher's Exact Test for Count Data
## 
## data:  contingency_table
## p-value < 2.2e-16
## alternative hypothesis: true odds ratio is not equal to 1
## 95 percent confidence interval:
##  12.93894 17.15664
## sample estimates:
## odds ratio 
##   14.89243 
## 
## 
## $contingency
##           unif.early.unif.rep !unif.early.unif.rep
## zld.down                  512                 2020
## !zld.down                 398                23398
```

```
fishing(class1 = (htable$res.zld.padj < thresh & htable$res.zld.FC < -1 * fcthresh), 
    class2 = peaks$unif.late, class1Name = "zld.down", class2Name = "unif.late")
```

```
## $fisher
## 
##  Fisher's Exact Test for Count Data
## 
## data:  contingency_table
## p-value < 2.2e-16
## alternative hypothesis: true odds ratio is not equal to 1
## 95 percent confidence interval:
##  0.06998126 0.25442343
## sample estimates:
## odds ratio 
##  0.1410623 
## 
## 
## $contingency
##           unif.late !unif.late
## zld.down         11       2521
## !zld.down       714      23082
```

```
fishing(class1 = (htable$res.zld.padj < thresh & htable$res.zld.FC > fcthresh), class2 = peaks$unif.late, 
    class1Name = "zld.up", class2Name = "unif.late")
```

```
## $fisher
## 
##  Fisher's Exact Test for Count Data
## 
## data:  contingency_table
## p-value = 3.445e-15
## alternative hypothesis: true odds ratio is not equal to 1
## 95 percent confidence interval:
##  2.708939 4.820937
## sample estimates:
## odds ratio 
##   3.641936 
## 
## 
## $contingency
##         unif.late !unif.late
## zld.up         59        608
## !zld.up       666      24995
```

I just sampled some of the comparisons. Bcd-dependence is enriched for `wt.early.late.rep` classes, as expected, not enriched in the ‘mutant’ version of this. Zld is also enriched in `wt.early.late.rep`. Zld-dependent regions are enriched in the set of uniform early and depleted from the uniform late classes. Interestingly, regions that go ‘up’ in Zelda (an often overlooked group) are enriched for regions that gain accessibility late. There’s something interesting there.

I want to go through all the different test permutations and plot a summary.

```
class2Names = c("unif.early.unif.rep", "unif.early.wt.rep", "unif.early.mut.rep", 
    "wt.early.late.rep", "mut.early.late.rep", "wt.early.constitutive", "mut.early.constitutive", 
    "unif.late", "wt.late", "mut.late")

bcd.down.tests = sapply(class2Names, function(x) fishing(class1 = (htable$res.bcd.padj < 
    thresh & htable$res.bcd.FC < -1 * fcthresh), class2 = mcols(peaks[, x])[, 1], 
    class1Name = "bcd.down", class2Name = x), simplify = FALSE)

bcd.up.tests = sapply(class2Names, function(x) fishing(class1 = (htable$res.bcd.padj < 
    thresh & htable$res.bcd.FC > fcthresh), class2 = mcols(peaks[, x])[, 1], class1Name = "bcd.up", 
    class2Name = x), simplify = FALSE)

zld.down.tests = sapply(class2Names, function(x) fishing(class1 = (htable$res.zld.padj < 
    thresh & htable$res.zld.FC < -1 * fcthresh), class2 = mcols(peaks[, x])[, 1], 
    class1Name = "zld.down", class2Name = x), simplify = FALSE)

zld.up.tests = sapply(class2Names, function(x) fishing(class1 = (htable$res.zld.padj < 
    thresh & htable$res.zld.FC > fcthresh), class2 = mcols(peaks[, x])[, 1], class1Name = "zld.up", 
    class2Name = x), simplify = FALSE)
```

```
odds.plotter = function(tests, title) {
    odds = lapply(tests, function(x) log2(x[[1]]$estimate))
    odds = do.call("c", odds)
    odds[is.infinite(odds)] = 0
    names(odds) = substring(names(odds), 1, (nchar(names(odds)) - 11))
    par(mar = c(4, 15, 1, 3))
    poscol = "tomato"
    negcol = "midnightblue"
    plotcols = rep(NA, length(odds))
    plotcols[which(odds > 0)] = poscol
    plotcols[which(odds <= 0)] = negcol
    plotcols = rev(plotcols)
    barplot(rev(odds), horiz = TRUE, las = 1, xlim = c(-8, 8), xaxt = "n", col = plotcols, 
        xlab = "log2(odds ratio)", main = title)
    # mtext(1, 'log2(odds ratio)', line = 2)
    axis(1, at = seq(-8, 8, length.out = 5))
}
```

```
odds.plotter(bcd.down.tests, "bcd-dependent")
```

```
odds.plotter(zld.down.tests, "zld-dependent")
```

```
odds.plotter(bcd.up.tests, "bcd-suppressed")
```

```
odds.plotter(zld.up.tests, "zld-suppressed")
```

# Incorporating Odd-paired ATAC data.

Because the group `wt.late` is significantly enriched for motifs for odd-paired (opa) we tested whether opa mutants had reduced chromatin accessibility at the NC14+72 timepoint. We can also produce DESeq tables for opa and repeat these analyses. The way this experiment was done, individual embryos from an opa/bal cross were collected and subjected to ATAC. Genotyping was performed post-hoc and genotypes were assigned on the basis of the distribution of recovered snps in the opa coding sequence (including the causative mutation).

```
# load the metadata
opameta = read.delim("~/Dropbox/HTseq/Analysis/opa_ATAC/171117_ATAC_library_prep_data.txt", 
    stringsAsFactors = FALSE, sep = "\t", header = TRUE)

load(opareadsfile1)  #allopen
load(opareadsfile2)  #allopen2
allopen = c(allopen, allopen2)

# put the data in the same order as the metadata list.
dataname = names(allopen)
dataname = substring(dataname, (nchar(dataname) - 7), nchar(dataname))

allopen = allopen[match(opameta[, 1], dataname)]

# for the opa analysis, we want to ensure that all of the Opa ChIP peaks are
# included in the analysis as well. There are some that weren't called peaks in
# the ATAC analysis. To do this, we'll create an object 'peaks.plus' that
# contains the reduction of the atac and opa ChIP peaks.

load(opaChIPpeaksfile)

p = peaks
mcols(p) = NULL
mcols(p) = data.frame(max = peaks$max)
op = opa.ChIP.peaks
mcols(op) = NULL
tm = start(opa.ChIP.peaks) + opa.ChIP.peaks$peak
mcols(op) = data.frame(max = tm)
peaks.plus = c(p, op)

names(peaks.plus) = NULL
peaks.plus = sort(peaks.plus)
peaks.plus = reduce(peaks.plus)

peaks.plus = peaks.plus[seqnames(peaks.plus) %in% c("chr2L", "chr2R", "chr3L", "chr3R", 
    "chr4", "chrX")]


opacounts = lapply(allopen, function(x) countOverlaps(peaks.plus, x))
opacounts = do.call("cbind", opacounts)

# generate the column data from the metadata file
ocoldata = opameta
rownames(ocoldata) = paste0(ocoldata[, 2], "_", ocoldata[, 3], "_", ocoldata[, 1])  # generate a vector that matches the prior names assigned to each ATAC sample
ocoldata = ocoldata[, -c(1:3)]  # lose the first three columns which is now redundant with the rownames
ocoldata = ocoldata[, c(7:8)]  # keep the columns that could be of potential interest in this analysis.

for (i in 1:ncol(ocoldata)) {
    ocoldata[, i] = as.factor(ocoldata[, i])
}  # convert all columns to 'factor'

# create an ordering vector:
orderer = c(which(ocoldata[, 2] == "wt"), which(ocoldata[, 2] == "het"), which(ocoldata[, 
    2] == "opa"))

# reorder the counts table:
opacounts = opacounts[, orderer]

# reorder the coldata table:
ocoldata = ocoldata[orderer, ]

# remove the hets:
opacounts = opacounts[, !ocoldata$zyg_genotype == "het"]
ocoldata = ocoldata[!ocoldata$zyg_genotype == "het", ]

# make sure the intended reference factor levels are the first factor levels
ocoldata[, 1] = relevel(ocoldata[, 1], ref = "pool_1")
ocoldata[, 2] = relevel(ocoldata[, 2], ref = "wt")
# The others are ok to leave as the minimum value

# check that everything is in register:
all(rownames(ocoldata) %in% colnames(opacounts))
```

```
## [1] TRUE
```

```
all(rownames(ocoldata) == colnames(opacounts))
```

```
## [1] TRUE
```

```
# initialize the DESeq dataset and process it:

opadds = DESeqDataSetFromMatrix(countData = opacounts, colData = ocoldata, design = ~zyg_genotype)
opadds = DESeq(opadds, parallel = TRUE)
resultsNames(opadds)  # check the output of the comparisons made based on the design above.
```

```
## [1] "Intercept"              "zyg_genotype_opa_vs_wt"
```

```
res.opa = results(opadds, contrast = c("zyg_genotype", "opa", "wt"), filterFun = ihw, 
    alpha = alphalevel)

# to recover the correspondence with the atac or the chip peaks table, we can
# filter the peaks.plus object for overlaps with either of the other peaks lists
in.atac = subjectHits(findOverlaps(peaks, peaks.plus))
in.chip = subjectHits(findOverlaps(opa.ChIP.peaks, peaks.plus))

otable = data.frame(baseMean = round(res.opa$baseMean[in.atac], 4), res.opa.FC = round(res.opa$log2FoldChange[in.atac], 
    4), res.opa.padj = res.opa$padj[in.atac])
otable$res.opa.padj[is.na(otable$res.opa.padj)] = 1

ochiptable = data.frame(baseMean = round(res.opa$baseMean[in.chip], 4), res.opa.FC = round(res.opa$log2FoldChange[in.chip], 
    4), res.opa.padj = res.opa$padj[in.chip])
ochiptable$res.opa.padj[is.na(ochiptable$res.opa.padj)] = 1
```

Volcano for opa.

```
volcanize(fc = otable$res.opa.FC, padj = otable$res.opa.padj, meanSig = otable$baseMean, 
    title = "opa, NC14+72", xlab.add.on = " (opa/wt)")
```

Class enrichment of significantly up or down regions.

```
opa.down.tests = sapply(class2Names, function(x) fishing(class1 = (otable$res.opa.padj < 
    thresh & otable$res.opa.FC < -1 * fcthresh), class2 = mcols(peaks[, x])[, 1], 
    class1Name = "opa.down", class2Name = x), simplify = FALSE)

opa.up.tests = sapply(class2Names, function(x) fishing(class1 = (otable$res.opa.padj < 
    thresh & otable$res.opa.FC > fcthresh), class2 = mcols(peaks[, x])[, 1], class1Name = "opa.up", 
    class2Name = x), simplify = FALSE)
```

```
odds.plotter(opa.down.tests, "opa-dependent")
```

```
odds.plotter(opa.up.tests, "opa-suppressed")
```

What fraction of the `wt.late` group can we account for with opa alone?

```
opa.down.tests["wt.late"]
```

```
## $wt.late
## $wt.late$fisher
## 
##  Fisher's Exact Test for Count Data
## 
## data:  contingency_table
## p-value < 2.2e-16
## alternative hypothesis: true odds ratio is not equal to 1
## 95 percent confidence interval:
##  52.21764 90.81568
## sample estimates:
## odds ratio 
##   68.89536 
## 
## 
## $wt.late$contingency
##           wt.late !wt.late
## opa.down      114      205
## !opa.down     208    25801
```

Within the set of sites that gain accessibility late specifically in the mutant, 35% of these sites require opa to gain chromatin accessibility.

## Maternal opa misexpression ATAC.

```
# load the metadata
tubmeta = read.delim(tubmetafile, stringsAsFactors = FALSE, sep = "\t", header = TRUE)

load(tubATACreads)  #tub.open 
load(wt13ATACreads)  #wt.open (does this overwrite a prior variable name?)
tubopen = c(wt.open, tub.open)
rm(list = c("tub.open", "wt.open"))

# put the data in the same order as the metadata list.
tubopen = tubopen[match(tubmeta[, 1], names(tubopen))]


tubcounts = lapply(tubopen, function(x) countOverlaps(peaks.plus, x))
tubcounts = do.call("cbind", tubcounts)

# generate the column data from the metadata file
shortgeno = rep(NA, nrow(tubmeta))
shortgeno[tubmeta$genotype %in% "w; HisGFP"] = "wt13"
shortgeno[tubmeta$genotype %in% "w; tub>opa/+; HisGFP/+"] = "tubopa"
rownames(tubmeta) = paste0(rownames(tubmeta), "_", shortgeno)
colnames(tubcounts) = paste0(colnames(tubcounts), "_", shortgeno)
tcoldata = data.frame(sample.name = tubmeta$sample.name, genotype = shortgeno)
rownames(tcoldata) = rownames(tubmeta)


for (i in 1:ncol(tcoldata)) {
    tcoldata[, i] = as.factor(tcoldata[, i])
}  # convert all columns to 'factor'

# create an ordering vector:
orderer = c(which(tcoldata[, 2] == "wt13"), which(tcoldata[, 2] == "tubopa"))

# reorder the counts table:
tubcounts = tubcounts[, orderer]

# reorder the coldata table:
tcoldata = tcoldata[orderer, ]

# make sure the intended reference factor levels are the first factor levels
tcoldata[, 2] = relevel(tcoldata[, 2], ref = "wt13")

# check that everything is in register:
all(rownames(tcoldata) %in% colnames(tubcounts))
```

```
## [1] TRUE
```

```
all(rownames(tcoldata) == colnames(tubcounts))
```

```
## [1] TRUE
```

```
# initialize the DESeq dataset and process it:

tubdds = DESeqDataSetFromMatrix(countData = tubcounts, colData = tcoldata, design = ~genotype)
tubdds = DESeq(tubdds, parallel = TRUE)
resultsNames(tubdds)  # check the output of the comparisons made based on the design above.
```

```
## [1] "Intercept"               "genotype_tubopa_vs_wt13"
```

```
res.tub = results(tubdds, contrast = c("genotype", "tubopa", "wt13"), filterFun = ihw, 
    alpha = alphalevel)

ttable = data.frame(baseMean = round(res.tub$baseMean[in.atac], 4), res.tub.FC = round(res.tub$log2FoldChange[in.atac], 
    4), res.tub.padj = res.tub$padj[in.atac])
ttable$res.tub.padj[is.na(ttable$res.tub.padj)] = 1
tchiptable = data.frame(baseMean = round(res.tub$baseMean[in.chip], 4), res.tub.FC = round(res.tub$log2FoldChange[in.chip], 
    4), res.tub.padj = res.tub$padj[in.chip])
tchiptable$res.tub.padj[is.na(tchiptable$res.tub.padj)] = 1
```

Volcano for tub>opa.

```
volcanize(fc = ttable$res.tub.FC, padj = ttable$res.tub.padj, meanSig = ttable$baseMean, 
    title = "tub>opa NC13 + 12", xlab.add.on = " (tub/wt)")
```

## Appending these comparisons to the ATAC peaks list

We have all these DESeq2 comparisons, and we want the data to be included in the peaks list. I have to be careful to make sure that none of the column names are redundant, and that it is clear what column belongs to what set. With the exception of the tub>opa comparison, it would make sense to order them by stage of the biological sample. To keep it readable, we should limit the adjusted p-values to four significant digits.

```
meta = mcols(peaks)

meta$res.wt.time.padj = signif(meta$res.wt.time.padj, 4)
meta$res.mut.time.padj = signif(meta$res.mut.time.padj, 4)
meta$res.12.geno.padj = signif(meta$res.12.geno.padj, 4)
meta$res.72.geno.padj = signif(meta$res.72.geno.padj, 4)

addendum = data.frame(bcd.zld.baseMean = htable$baseMean, res.bcd.FC = htable$res.bcd.FC, 
    res.bcd.padj = signif(htable$res.bcd.padj, 4), res.zld.FC = htable$res.zld.FC, 
    res.zld.padj = signif(htable$res.zld.padj, 4), opa.baseMean = otable$baseMean, 
    res.opa.FC = otable$res.opa.FC, res.opa.padj = signif(otable$res.opa.padj, 4), 
    tub.baseMean = ttable$baseMean, res.tub.FC = ttable$res.tub.FC, res.tub.padj = signif(ttable$res.tub.padj, 
        4))

meta = cbind(meta, addendum)

mcols(peaks) = meta
```

And the opa ChIP peaks.

```
meta = mcols(opa.ChIP.peaks)
max = meta$peak + start(opa.ChIP.peaks)
score = opa.ChIP.peaks$q.value

addendum = data.frame(score = score, max = max, opa.baseMean = ochiptable$baseMean, 
    res.opa.FC = ochiptable$res.opa.FC, res.opa.padj = signif(ochiptable$res.opa.padj, 
        4), tub.baseMean = tchiptable$baseMean, res.tub.FC = tchiptable$res.tub.FC, 
    res.tub.padj = signif(tchiptable$res.tub.padj, 4))

mcols(opa.ChIP.peaks) = addendum
```

## Packaging the annotated peaks lists for publication

To publish the annotated peaks list, we want to clean up the metadata a little bit and save both an R-compatible and tabular object. This is not evaluated in the markdown, but is here as a record.

```
seqlevels(peaks) = seqlevels(Dmelanogaster)
seqinfo(peaks) = seqinfo(Dmelanogaster)
seqlevels(peaks) = seqlevelsInUse(peaks)
genome(peaks) = "dm6"

save(peaks, file = "~/Dropbox/Blythe Lab/Patternless Paper/Final_Publication_Datasets/Annotated_ATAC_Peaks_Genomic_Ranges.R")

seqlevels(opa.ChIP.peaks) = seqlevels(Dmelanogaster)
seqinfo(opa.ChIP.peaks) = seqinfo(Dmelanogaster)
seqlevels(opa.ChIP.peaks) = seqlevelsInUse(opa.ChIP.peaks)
genome(opa.ChIP.peaks) = "dm6"

save(opa.ChIP.peaks, file = "~/Dropbox/Blythe Lab/Patternless Paper/Final_Publication_Datasets/Annotated_Opa_ChIP_Peaks_Genomic_Ranges.R")
```

```
sessionInfo()
```

```
## R version 3.5.2 (2018-12-20)
## Platform: x86_64-apple-darwin15.6.0 (64-bit)
## Running under: macOS Mojave 10.14
## 
## Matrix products: default
## BLAS: /Library/Frameworks/R.framework/Versions/3.5/Resources/lib/libRblas.0.dylib
## LAPACK: /Library/Frameworks/R.framework/Versions/3.5/Resources/lib/libRlapack.dylib
## 
## locale:
## [1] en_US.UTF-8/en_US.UTF-8/en_US.UTF-8/C/en_US.UTF-8/en_US.UTF-8
## 
## attached base packages:
##  [1] grid      parallel  stats4    stats     graphics  grDevices utils    
##  [8] datasets  methods   base     
## 
## other attached packages:
##  [1] zoo_1.8-4                                
##  [2] TxDb.Dmelanogaster.UCSC.dm6.ensGene_3.4.4
##  [3] GenomicFeatures_1.34.7                   
##  [4] AnnotationDbi_1.44.0                     
##  [5] BSgenome.Dmelanogaster.UCSC.dm6_1.4.1    
##  [6] BSgenome_1.50.0                          
##  [7] rtracklayer_1.42.2                       
##  [8] Biostrings_2.50.2                        
##  [9] XVector_0.22.0                           
## [10] Gviz_1.26.5                              
## [11] ggplot2_3.2.0                            
## [12] IHW_1.10.1                               
## [13] DESeq2_1.22.2                            
## [14] SummarizedExperiment_1.12.0              
## [15] DelayedArray_0.8.0                       
## [16] BiocParallel_1.16.6                      
## [17] matrixStats_0.54.0                       
## [18] Biobase_2.42.0                           
## [19] GenomicRanges_1.34.0                     
## [20] GenomeInfoDb_1.18.2                      
## [21] IRanges_2.16.0                           
## [22] S4Vectors_0.20.1                         
## [23] BiocGenerics_0.28.0                      
## 
## loaded via a namespace (and not attached):
##  [1] ProtGenerics_1.14.0       bitops_1.0-6             
##  [3] bit64_0.9-7               RColorBrewer_1.1-2       
##  [5] progress_1.2.0            httr_1.4.0               
##  [7] tools_3.5.2               backports_1.1.3          
##  [9] R6_2.4.0                  rpart_4.1-13             
## [11] Hmisc_4.2-0               DBI_1.0.0                
## [13] lazyeval_0.2.2            colorspace_1.4-1         
## [15] nnet_7.3-12               withr_2.1.2              
## [17] tidyselect_0.2.5          gridExtra_2.3            
## [19] prettyunits_1.0.2         curl_3.3                 
## [21] bit_1.1-14                compiler_3.5.2           
## [23] fdrtool_1.2.15            formatR_1.6              
## [25] htmlTable_1.13.1          labeling_0.3             
## [27] slam_0.1-45               scales_1.0.0             
## [29] checkmate_1.9.1           genefilter_1.64.0        
## [31] stringr_1.4.0             digest_0.6.18            
## [33] Rsamtools_1.34.1          foreign_0.8-71           
## [35] rmarkdown_1.13            dichromat_2.0-0          
## [37] base64enc_0.1-3           pkgconfig_2.0.2          
## [39] htmltools_0.3.6           lpsymphony_1.10.0        
## [41] ensembldb_2.6.7           htmlwidgets_1.3          
## [43] rlang_0.3.4               rstudioapi_0.10          
## [45] RSQLite_2.1.1             acepack_1.4.1            
## [47] dplyr_0.8.1               VariantAnnotation_1.28.13
## [49] RCurl_1.95-4.12           magrittr_1.5             
## [51] GenomeInfoDbData_1.2.0    Formula_1.2-3            
## [53] Matrix_1.2-15             Rcpp_1.0.1               
## [55] munsell_0.5.0             stringi_1.4.3            
## [57] yaml_2.2.0                zlibbioc_1.28.0          
## [59] blob_1.1.1                crayon_1.3.4             
## [61] lattice_0.20-38           splines_3.5.2            
## [63] annotate_1.60.1           hms_0.4.2                
## [65] locfit_1.5-9.1            knitr_1.22               
## [67] pillar_1.3.1              geneplotter_1.60.0       
## [69] biomaRt_2.38.0            XML_3.98-1.19            
## [71] glue_1.3.1                evaluate_0.13            
## [73] biovizBase_1.30.1         latticeExtra_0.6-28      
## [75] data.table_1.12.0         gtable_0.3.0             
## [77] purrr_0.3.2               assertthat_0.2.1         
## [79] xfun_0.5                  xtable_1.8-3             
## [81] AnnotationFilter_1.6.0    survival_2.43-3          
## [83] tibble_2.1.1              GenomicAlignments_1.18.1 
## [85] memoise_1.1.0             cluster_2.0.7-1
```
