# Supplementary material for: Zygotic pioneer factor activity of Odd-paired/Zic is necessary for late function of the Drosophila segmentation network
Source: eLife. 2020 Apr 29;9:e53916. doi: 10.7554/eLife.53916 (PMC7190358; doi:10.7554/eLife.53916)
Supplement: Transparent reporting form [file elife-53916-transrepform.docx]

***eLife’s* transparent reporting form**

We encourage authors to provide detailed information *within their submission* to facilitate the interpretation and replication of experiments. Authors can upload supporting documentation to indicate the use of appropriate reporting guidelines for health-related research (see [EQUATOR Network](http://www.equator-network.org/%20)), life science research (see the [BioSharing Information Resource](https://biosharing.org/)), or the [ARRIVE guidelines](http://www.plosbiology.org/article/info:doi/10.1371/journal.pbio.1000412) for reporting work involving animal research. Where applicable, authors should refer to any relevant reporting standards documents in this form.

If you have any questions, please consult our Journal Policies and/or contact us: [editorial@elifesciences.org](mailto:editorial@elifesciences.org).

**Sample-size estimation**

- You should state whether an appropriate sample size was computed when the study was being designed
- You should state the statistical method of sample size computation and any required assumptions
- If no explicit power analysis was used, you should describe how you decided what sample (replicate) size (number) to use

Please outline where this information can be found within the submission (e.g., sections or figure legends), or explain why this information doesn’t apply to your submission:

The rationale for ATAC-seq replicate number choice is described at the end of the “ATAC-seq” section of the Materials and Methods.

**Replicates**

- You should report how often each experiment was performed
- You should include a definition of biological versus technical replication
- The data obtained should be provided and sufficient information should be provided to indicate the number of independent biological and/or technical replicates
- If you encountered any outliers, you should describe how these were handled
- Criteria for exclusion/inclusion of data should be clearly stated
- High-throughput sequence data should be uploaded before submission, with a private link for reviewers provided (these are available from both GEO and ArrayExpress)

Please outline where this information can be found within the submission (e.g., sections or figure legends), or explain why this information doesn’t apply to your submission:

The definition of a ‘biological replicate’ is included in the “ATAC-seq” section of the Materials and Methods. No outliers were encountered. All recovered sequencing data were included. A submission to GEO is currently in process. We have made processed data available on Github (<https://github.com/sblythe/Patternless_ATAC>) including a step-by-step markdown that demonstrates the data analysis procedure, and reproduces many of the critical figures of the paper. We will communicate to the Editor once the GEO submission is complete and a private link has been generated.

**Statistical reporting**

- Statistical analysis methods should be described and justified
- Raw data should be presented in figures whenever informative to do so (typically when N per group is less than 10)
- For each experiment, you should identify the statistical tests used, exact values of N, definitions of center, methods of multiple test correction, and dispersion and precision measures (e.g., mean, median, SD, SEM, confidence intervals; and, for the major substantive results, a measure of effect size (e.g., Pearson's r, Cohen's d)
- Report exact p-values wherever possible alongside the summary statistics and 95% confidence intervals. These should be reported for all key questions and not only when the p-value is less than 0.05.

Please outline where this information can be found within the submission (e.g., sections or figure legends), or explain why this information doesn’t apply to your submission:

Any plots using standard deviation have that fact clearly stated in the axis label and/or figure legend (e.g., Figure 5B). Any plots using statistical tests to determine gene set enrichment (e.g., Figure 5 supplement 2) clearly indicate the test and the sample sizes in either the figure itself or in the figure legend. Exact p-values are provided. For DESeq2 analysis, major substantive results are summarized in the Results section text, technical detail is provided in the Materials and Methods, and an extensive Markdown document of the analysis is provided at the lab GitHub page.

(For large datasets, or papers with a very large number of statistical tests, you may upload a single table file with tests, Ns, etc., with reference to sections in the manuscript.)

**Group allocation**

- Indicate how samples were allocated into experimental groups (in the case of clinical studies, please specify allocation to treatment method); if randomization was used, please also state if restricted randomization was applied
- Indicate if masking was used during group allocation, data collection and/or data analysis

Please outline where this information can be found within the submission (e.g., sections or figure legends), or explain why this information doesn’t apply to your submission:

Samples were not collected blind, except in the case of the *opa* zygotic mutant ATAC experiment, where the experimenter was blind to the zygotic genotype of the sample until following recovery of sequencing data. Randomization was not performed.

**Additional data files (“source data”)**

- We encourage you to upload relevant additional data files, such as numerical data that are represented as a graph in a figure, or as a summary table
- Where provided, these should be in the most useful format, and they can be uploaded as “Source data” files linked to a main figure or table
- Include model definition files including the full list of parameters used
- Include code used for data analysis (e.g., R, MatLab)
- Avoid stating that data files are “available upon request”

Please indicate the figures or tables for which source data files have been provided:

Annotated peaks lists are provided as additional data files. A markdown document highlighting how critical analytical steps were performed is provided as an additional data file.
